# Psychometric evaluation of the healthy aging activity engagement scale

**DOI:** 10.3389/fpubh.2022.986666

**Published:** 2022-10-10

**Authors:** Tingting Lu, Linghui Kong, Huijun Zhang

**Affiliations:** Department of Nursing, Jinzhou Medical University, Jinzhou, China

**Keywords:** aged, healthy lifestyle, disease prevention, healthy aging, factor analysis

## Abstract

**Objective:**

The aim of this study was to translate the Healthy Aging Activity Engagement Scale (HAAE) into Chinese and validate its psychometric properties in the middle-aged and elderly population.

**Methods:**

A total of 424 middle-aged and elderly people were recruited from China's Jiangsu Province, Liaoning Province, Shandong Province, and Heilongjiang Province. Cronbach's α co-efficient, split-half reliability, and test-retest reliability were used to evaluate the reliability of the translated scale. Expert consultation was used to evaluate the content validity of the translated scale. Exploratory factor analysis (EFA) and confirmatory factor analysis (CFA) were used to evaluate the structural validity of the scale.

**Results:**

The Cronbach's α co-efficient of the Chinese version of HAAE was 0.965 and the Cronbach's α co-efficient of the dimensions ranged from 0.898 to 0.957. The split-half reliability was 0.807, and the test-retest reliability was 0.850. The content validity index of the scale (S-CVI) was 0.969. A total of three common factors were extracted from the EFA. The CFA validated the explored 3-factor structure, and the indicators were fitted well (χ^2^/df = 1.393, comparative fit index = 0.982, goodness- of- fit index = 0.911. Tucker-Lewis Index = 0.981 and root mean square error of approximation = 0.030).

**Conclusion:**

The translated Chinese version of HAAE had suitable reliability and validity in the middle-aged and elderly population. The translated scale will be used to evaluate the level of healthy aging among middle-aged and elderly people in Chinese mainland. Furthermore, it also can provide some health advice for clinical patients.

## Introduction

Aging of population is not only a problem in developed countries, but also in some developing countries. According to China's latest census, people aged 60 and above accounted for 18.7 % of the total population, of which 13.5% were aged 65 and above (1). With the increasing degree of population aging, Chronic diseases account for more prominent proportions of the elderly population. The occurrence of chronic diseases greatly affects their quality of life and increases the financial burden on the family. At the same time, studies have shown that older people are able to benefit from an active lifestyle (2). Furthermore, psychological state has also become an important factor affecting health. Some scholars have pointed out that psychological stress has also become a contributing factor to the disease, and studies have shown that stress increases the incidence and severity of cardiovascular diseases, diabetes and obesity (3, 4). Combining a holistic lifestyle of healthy eating, regular exercise and stress reduction will improve quality of life and reduce cardiovascular and the occurrence of cancer (5, 6). Moreover, healthy lifestyle should not be vigorously promoted among older people, but should be promoted as early as possible. There are a number of other studies that have shown that obesity in the middle-aged population increases the incidence of cardiovascular disease as well as dementia (7, 8). The incidence of stroke and kidney disease in middle-aged people is also rising (9). Therefore, for middle-aged people, it is necessary to participate in a healthy aging lifestyle as soon as possible.

A healthy aging lifestyle are primarily about engaging in exercise, cognitive and social engagement, and reducing stress. Engaging in more physical activity can delay cognitive decline (10, 11). Physical capability and activities at older ages is not only an important aspect of a healthy life but also reduces the risk of pre-mature death (12). Moreover, broader social engagement can make older people feel more rewarded in their lives and it can reduce the risk of frailty of the elderly (12, 13). Meanwhile, the concept of healthy aging is affected by stress; stress increases the incidence of a range of chronic diseases later in life, such as CVD (14–17). Therefore, in the process of healthy aging, we must not only pay attention to physical exercise and reasonable diet structure, but also pay attention to some factors related to psychology (such as cognitive changes, stress, etc.).

Some questionnaires are able to assess the level of healthy behaviors in certain specific areas (such as stress, physical health, diet) (18). However, there is currently no scale that can comprehensively assess the level of individual participation in multidimensional healthy aging, especially among middle-aged and elderly people in China. Professor Maureen Schmitter-Edgecombe used traditional measurement analysis and Rasch modeling techniques to develop a Healthy Aging Activity Engagement scale (HAAE) and conducted validation in 2017 (19). In contrast to traditional scales, the HAAE scale is no longer a single assessment of a particular aspect of health behavior involvement; It comprehensively evaluates the healthy aging behavior of middle-aged and elderly people from multiple perspectives.

This study aimed to translate the HAAE into Chinese and validate its psychometric properties. To provide a tool for measuring the level of engagement in healthy aging among middle-aged and elderly Chinese population.

## Methods

### Study design and participants

The multicenter cross-sectional study was conducted in China from December 2021 to February 2022. A total of 424 participants were recruited using the convenient sampling method, which comes from China's Jiangsu Province, Liaoning Province, Shandong Province and Heilongjiang Province.Participants were selected from community. The sample size required at least three participants for each factor. If possible, it also can obtain larger sample sizes (18). In this study, to ensure the accuracy of exploratory factors analysis and confirmatory factor analysis, a minimum of 10 participants were required for each item. Included participants should meet the following criteria: (1) Age ≥ 45 years. (2) The length of time of residence in the local area≥6 months. (3) Participants were able to understand the questionnaire and completed it independently. (4) People who gave informed consent and voluntarily participated in this study. People with severe physical and mental illnesses were excluded, such as impaired consciousness, severe physical activity disorder, advanced malignancy, and inability to take care of themselves.

### Instruments

#### General demographic characteristics questionnaire

A questionnaire on general demographic characteristics was designed by reading the literature related to this study prior to conducting the survey. It mainly includes five items: age, gender, education level, occupational status, and self-assessment lifestyle health.

#### Healthy aging activity engagement scale

This study used the Healthy Aging Activity Engagement Scale developed by Professor Maureen and others in 2017 (19). The scale consists mainly of 32 items. Scoring was made using the Likert 5-level scoring method, with scores ranging from 1 to 5. The scoring criteria were as follows: 1 = Strongly disagree, 2 = disagree, 3 = Somewhat agree, 4 = agree, 5 = Strongly agree. The scale mainly evaluated the engagement of middle-aged and elderly people in healthy aging from three dimensions: biologic health, social and cognitive strategies and health safeguard behaviors. A higher score indicated a higher level of participation in healthy aging. Both the reliability and validity of the translated scale were within acceptable limits.

### Procedures

#### Translation and cultural adaptation

With the consent of Professor Maureen, the scale was translated into a Chinese version and cultural adaptation was implemented. The original scale was translated using the Brislin double-translation model (20). First, two Master's Chinese students majoring in English translated the scale into Chinese. After negotiation, a Chinese version of the scale was finally formed. Then, two native English-speaking Chinese teachers reverse-translate the translated content without reading the original scale. Three nursing professors were invited to assess whether the translated items were in line with Chinese language expression habits. The researchers made modifications based on the results of the expert review. Finally, a scale suitable for measuring the level of participation in healthy aging activities among middle-aged and elderly people in China was formed. Twenty middle-aged and elderly people were selected for pre-survey to validate whether the items for the translated scale were easy to read and understand. The results showed that it took about 6 min to complete the scale and the content can be understood.

#### Data collection procedure

The researchers collected questionnaires from four provinces, respectively. A total of 450 middle-aged and elderly people were recruited in the community using the convenient sampling method. Four hundred and forty people volunteered to participate in, cross-sectional surveys were used on these populations. All questionnaires were completed anonymously. After excluding invalid questionnaires, 424 questionnaires were collected. The effective recovery rate of the questionnaires was 96.36%.

### Data analysis procedure

#### Items analysis

In items analysis, divide the total score of the scale into two parts: the first 27%was high-score group and the last 27% was poor-score group. By comparing the relationship between the two to judge the reliability and discernment of the translated items. Using the critical ratio to judge whether the difference between the items was statistically significant. Cronbach's α co-efficient if item deleted was used to judge whether items for the translated scale needed to be deleted.

#### Reliability analysis

Reliability refers to the consistency or repeatability of a measurement (21). The internal consistency of the scale was evaluated using the Cronbach's α co-efficient and split-half reliability. Items were divided into equal groups according to the parity principle and calculate the relationship between the two to represent the split-half reliability. The time interval between two measurements in the elderly is 2 weeks (22). After 2 weeks, a total of 40 people were selected from the original population and measured again to reflect the test-retest reliability. It reflected the consistency of the two tests.

#### Validity analysis

The evaluation of the content validity was assessed by seven experts using the Delphi Expert Consultation method. Its evaluation results were expressed in terms of the content validity index of the items (I-CVI) and the content validity index of the scale (S-CVI). Each item was divided into 4 levels (from inappropriate to appropriate), which was scored separately by each expert. The value of I-CVI is equal to the number of experts scored 3 or 4 points divided by the total number of experts participating in the score. S-CVI is the average of the I-CVI value for each item.

The EFA and CFA were used to explore and verify the potential factor structure of the scale, respectively. The 424 participants were divided into two groups of the same number according to the principle of randomization. Each group of 212 people were tested by EFA and CFA, respectively. The scale is suitable for factor analysis only when the KMO > 0.6 and the Bartlett spherical test was statistically significant (*P* < 0.05). Amos (23.0) software was used to validate the consistency of the model structure with the explored factor structure. CMIN/DF, comparative fit index (CFI), goodness- of- fit index (GFI) and Tucker- Lewis Index (TLI) were used to represent the fit of the model. The closer the CMIN/DF value is to 0, the better fit of the model (23, 24). When the values of CFI, GFI, and TLI are ≥ 0.9, it means that the model fits well (23, 25). RMSEA is used to evaluate the degree of unfitting of a model, and the closer its value is to 0, the better its fit (23, 25).

### Ethical approval

Each participant completed an informed consent form. The information in each questionnaire was protected. Moreover, This study was approved by the Ethics Committee of the Jinzhou Medical University and the process followed the code of ethics provided by the Ethics Committee.

## Results

### Demographic information

A total of 424 participants who met the criteria were included: 205 females (48.3%) and 219 males (51.7%). The elderly group aged 60 and above accounted for 62.7%.Middle-aged population accounted for 37.3%. 60.4% of the population was in retirement. Only 13.7 percent rated themselves as having a poor lifestyle. The remaining demographic information can be found in Table 1.

**Table 1:** General demography data (*n* = 424).

**Items**	**Group**	***n***	**%**
**Age**	45–50	50	11.8
	50–55	38	9.0
	55–60	70	16.5
	≥60	266	62.7
**Sex**	Male	219	51.7
	Female	205	48.3
**Educational level**	Primary school and below	145	34.2
	Junior high school	172	0.6
	High school and junior college	99	23.3
	University and above	8	1.9
**Occupation**	Work	114	26.9
	Retire	256	60.4
	Unemployment	54	12.7
**Lifestyle**	Poor	58	13.7
	Not bad	183	43.2
	Good	183	43.2

### Item analysis

The critical ratio was used to indicate the degree of differentiation of the item. When critical ratio ≥ 3, it means that the item is better distinguished. In this translated scale, the critical ratio of the 32 items were ranged from 8.354 to 15.468. It indicated that the differentiation of each item was within acceptable range. The Pearson Correlation Analysis method was used to analyze the relevance between the items and the total score. The relationship between the items and the scale was moderately correlated (*r* = 0.547–0.688, *P* < 0.05). After the items were deleted, the Cronbach's α value for each item ranged from 0.964–0.965. The value of each item was lower than the total Cronbach's α value of the scale (0.965). Therefore, each item can be accepted without deletion.

### Reliability analysis

The total Cronbach's α co-efficient of the scale was 0.965. Cronbach's α co-efficient for each dimension ranged from 0.898 to 0.957. Moreover, the scale had a split-half reliability of 0.807. After 2 weeks, 40 people were randomly selected to obtain a test-retest reliability of 0.850. It can be concluded that the translated scale had a suitable reliability (Table 2).

**Table 2:** Reliability analysis for Chinese version of the HAAE.

**The scale and its dimension**	**Cronbach's Alpha**	**Split-half reliability**	**Test-retest reliability**
The HAAE	0.965	0.807	0.850
Biological health	0.957		
Social and cognitive strategies	0.954		
Health safeguard behaviors	0.898		

### Validity analysis

#### Content validity analysis

The content validity of the translated scale was evaluated by 7 experts. Each item was separately scored by each expert. The range of the I-CVI was 0.857–1.000 and the value of S-ICV was calculated to be 0.969. The translated scale had the suitable content reliability (Table 3).

**Table 3:** Item-level CVI for the Chinese version of the HAAE.

**HAAE items**	**Expert1**	**Expert2**	**Expert3**	**Expert4**	**Expert5**	**Expert6**	**Expert7**	**I-CVI**
1	2	3	3	3	3	3	3	0.857
2	3	4	4	4	3	4	4	1.000
3	4	3	2	4	4	4	4	0.857
4	4	4	3	3	4	3	3	1.000
5	3	4	4	4	4	3	3	1.000
6	3	4	4	2	4	3	3	0.857
7	3	3	4	3	3	2	3	0.857
8	3	4	4	3	4	3	4	1.000
9	4	4	4	4	3	3	3	1.000
10	3	4	3	3	3	4	4	1.000
11	4	4	4	4	3	4	3	1.000
12	4	3	4	3	3	4	3	1.000
13	4	3	4	4	3	4	3	1.000
14	3	3	4	4	4	3	3	1.000
15	4	3	3	3	3	4	4	1.000
16	4	4	4	4	4	3	3	1.000
17	3	3	3	4	4	4	3	1.000
18	4	4	4	3	4	3	4	1.000
19	3	4	3	4	3	3	4	1.000
20	3	4	2	3	3	3	3	0.857
21	3	3	4	3	4	3	3	1.000
22	3	3	3	3	4	4	4	1.000
23	3	3	4	4	4	4	3	1.000
24	3	3	3	2	4	4	3	0.857
25	3	4	4	3	4	3	4	1.000
26	3	4	4	4	3	3	4	1.000
27	3	3	3	4	3	4	3	1.000
28	3	4	4	3	4	3	4	1.000
29	4	3	4	3	4	3	4	1.000
30	4	3	4	3	3	4	3	1.000
31	3	4	3	4	3	4	4	1.000
32	3	2	4	3	4	3	3	0.857

#### Exploratory factor analysis

**Figure 1:**
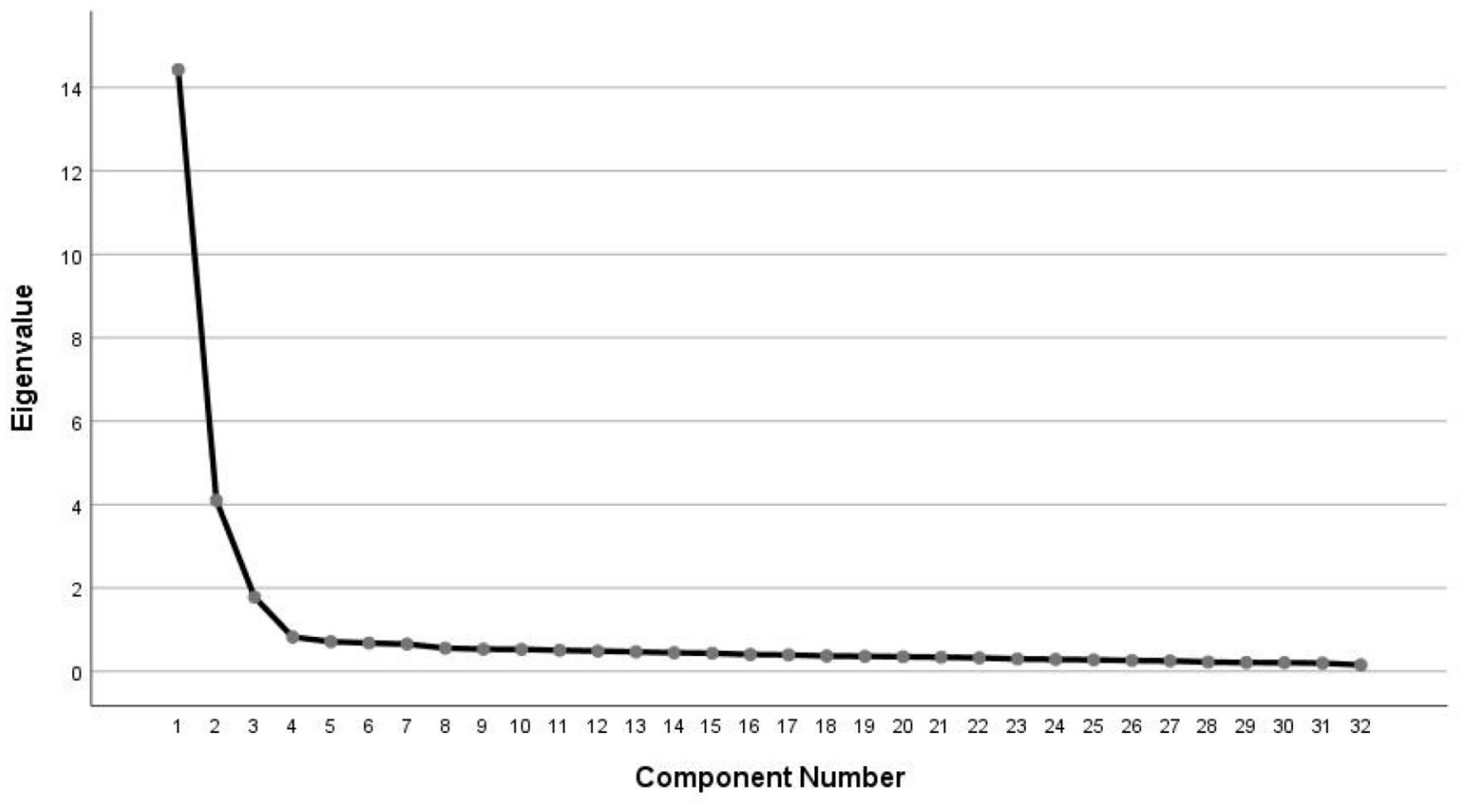
Screen plot of exploratory factor analysis for Chinese version of the HAAE.

In this study, KMO = 0.960, Bartlett's spherical test was statistically significant (χ^2^ = 4,819.743; *P* < 0.001). This results indicated that the translated scale was suitable for factor structure analysis. A total of three factors with eigenvalues > 1 were extracted and a total of 63.448% of the data discrepancies were explained. Screen plot (Figure 1) further confirmed the existence of the 3-factor structure. Furthermore, the results of EFA showed that each factor load was > 0.4. Therefore, all items were retained. The specific results can be found in Table 4.

**Table 4:** Factor loadings of exploratory factor analysis for the healthy aging activity engagement scale.

**Item**	**Factor1**	**Factor2**	**Factor3**
1	0.781	–	–
2	0.735	–	–
3	0.698	–	–
4	0.732	–	–
5	0.729	–	–
6	0.701	–	–
7	0.744		–
8	0.771		–
9	0.774		–
10	0.727		–
11	0.758		–
12	0.744		–
13	0.658		
14	0.720		
15	0.699		
16	–	0.758	–
17	–	0.734	–
18	–	0.764	–
19	–	0.779	–
20	–	0.797	–
21	–	0.770	–
22	–	0.760	–
23	–	0.748	–
24	–	0.784	–
25	–	0.773	–
26	–	0.703	–
27	–	–	0.708
28	–	–	0.713
29	–	–	0.748
30	–	–	0.733
31	–	–	0.651
32	–	–	0.721

### Confirmatory factor analysis

Confirmatory factor analysis was used to validate whether the relationship between each item and the factor is consistent with the hypothesis. In this study, the results of the validation showed that the fittings were good. The values of the indicators were: χ^2^/df = 1.393, CFI = 0.982, GFI = 0.911. TLI = 0.981, and RMSEA = 0.030. It showed that the translated scale factors correspond well to the corresponding items.

## Discussion

Healthy behavior is an important aspect of healthy aging (24). There are indeed some scales that assess healthy behavior of older adults (26, 27), But they were limited to a specific aspect, such as exercise. There is currently no tool that can measure the level of participation of middle-aged and elderly populations in healthy aging from multiple angles. The HAAE scale applied not only to the elderly, but also to middle-aged people. The development of the disease is showing a trend of rejuvenation. Similarly, Middle-aged people are also at risk of aging, Participation in healthy aging is imminent. Furthermore, the application of the HAAE scale in middle-aged people can greatly promote the dissemination of healthy aging knowledge, let them know the importance of preventing the occurrence of diseases as soon as possible, and reduce the occurrence of diseases after they enter old age. Therefore, this is an important tool for both the elderly and middle-aged to measure their own health participation.

The translation of the scale followed the Brislin double-translation model (20). The process mainly consists of two parts: translation and reverse-translation. In order to ensure the translated statement was simple, clear and easy to understand, the “in the past seven days” in each item was adjusted in the description at the head of the table. In addition, the “4 servings (1½−1 cup per serving) of fruits and vegetables” mentioned in the original scale item 4, In order to localize its expression, it was finally changed to “400 g fruits and vegetables” through literature review and expert correspondence. Three nursing specialists were invited to review the items of the translated scale. Finally, the items in the Chinese version of the HAAE scale were concise and easy to understand. By comparing the change in reliability after the deletion of items, it was concluded that 32 items were acceptable.

In this study, the reliability and validity of the Chinese version of the HAAE scale were examined. The I-CVI and S-CVI of the translated scale were higher than reference value (28). The three-factor structure extracted by EFA was consistent with the original scale (18), which can explain 63.448% of the variation and each item factor load was > 0.4.

At the same time, the CFA validate the degree of fit of the scale, the correlation co-efficients between the latent variables were all within the acceptable range (< 0.7), and the distinction between the dimensions was good. The translated scale had a Cronbach's α co-efficient of 0.965, which was higher than the original scale (18). In addition, both split-half reliability and test–retest reliability were acceptable (>0.8), and the translated scale had suitable stability.

## Limitations

This study had the following limitations: Firstly, there was no distinction between middle-aged and elderly people. Because of the influencing factors of the level of engagement in healthy aging activities in the two populations were different. Secondly, because of only selecting people who meet the requirements of some areas. There is some bias in this study in population selection.

## Conclusions

The translated HAAE scale had good reliability and validity. It was well used among middle-aged and elderly people in China after cultural adaption. Aging is the trend of Chinese population development. It can provide a measurement tool for the middle-aged and elderly population in China. The HAAE scale can be used to measure the extent to which they are involved in a healthy lifestyle. In addition, it can strengthen the health awareness of middle-aged and elderly people and promote the spread of healthy aging patterns.

## Implications

Healthy lifestyle is particularly important in the middle-aged and elderly population, which are related to the incidence of future diseases and the quality of life in later years. Therefore, it is very meaningful to assess their level of engagement in healthy aging. The translated HAAE scale will be used to assess the level of engagement in healthy aging in Chinese middle-aged and elderly population and make targeted recommendations.

## Data availability statement

The original contributions presented in the study are included in the article/supplementary material, further inquiries can be directed to the corresponding author.

## Author contributions

TL completed the writing of this article. HZ put forward important revision suggestions and made corresponding modifications for this article. LK played a key role in part of the data collection. All authors contributed to the article and approved the submitted version.

## Conflict of interest

The authors declare that the research was conducted in the absence of any commercial or financial relationships that could be construed as a potential conflict of interest.

## Publisher's note

All claims expressed in this article are solely those of the authors and do not necessarily represent those of their affiliated organizations, or those of the publisher, the editors and the reviewers. Any product that may be evaluated in this article, or claim that may be made by its manufacturer, is not guaranteed or endorsed by the publisher.
